# A Strategy to Reduce Heart Failure Readmissions and Inpatient Costs

**DOI:** 10.14740/cr384w

**Published:** 2015-02-09

**Authors:** Jill Howie-Esquivel, Maureen Carroll, Eileen Brinker, Helen Kao, Steven Pantilat, Karen Rago, Teresa De Marco

**Affiliations:** a Department of Physiological Nursing, University of California, San Francisco, CA, USA; b University of California San Francisco Medical Center, San Francisco, CA, USA

**Keywords:** Readmission, Heart failure, Disease management

## Abstract

**Background:**

The objective of this study was to evaluate the effect of a disease management intervention on rehospitalization rates in hospitalized heart failure (HF) patients.

**Methods:**

Patients treated with the TEACH-HF intervention that included Teaching and Education, prompt follow-up Appointments, Consultation for support services, and Home follow-up phone calls (TEACH-HF) from January 2010 to January 2012 constituted the intervention group (n = 548). Patients treated from January 2007 to January 2008 constituted the usual care group (n = 485).

**Results:**

Group baseline characteristics were similar with 30-day readmission rates significantly different (19% usual care vs. 12% for the intervention respectively (P = 0.003)). Patients in the usual care group were 1.5 times more likely to be hospitalized (95% CI: 1.2 - 1.9; P = 0.001) compared to the intervention group. A savings of 641 bed days with potential revenue of $640,000 occurred after TEACH-HF.

**Conclusions:**

The TEACH-HF intervention was associated with significantly fewer hospital readmissions and savings in bed days.

## Introduction

Heart failure (HF) is the most common discharge diagnosis among Medicare beneficiaries and the most common cause of hospital readmissions [1-3]. HF is also the most costly condition and accounts for more than $39 billion per year in healthcare expenditures [4]. HF is characterized by episodic exacerbations that often require hospitalization alternating with periods of medical stability. The goal of HF care is to manage symptoms in the outpatient setting to prevent HF hospitalizations as such episodes are costly and associated with increased morbidity and mortality. Recent changes to Medicare reimbursement impose financial penalties when patients with HF are readmitted within 30 days under the assumption that many readmissions are avoidable. Hospital admissions are avoidable when increased adherence to medication and diet, improved social support and greater access to medical assistance are facilitated [5-7]. The best means to provide better quality of care to HF patients to avoid rehospitalization is uncertain; however, key elements for high quality care may be identifiable and applied to all HF programs. HF patients are faced with managing a complex and progressive medical problem (i.e. HF) making the issue of hospital readmission challenging [7]. Given the increased morbidity, mortality, costs and penalties associated with HF readmissions, health care providers are seeking effective and reproducible interventions to reduce hospital readmissions.

HF disease management programs can include interventions that range from telephone calls to home visits with intensive technology-based monitoring and intervention systems [5-10]. Some of these programs have demonstrated improvements in patient outcomes; however, variability in the interventions has made it difficult to discern the key elements associated with improved outcomes [10]. In addition, intensive programs do not necessarily provide better outcomes and may increase associated personnel and administrative costs [11, 12]. Evaluation of such programs needs to be understood within: 1) their context or setting; 2) the health system in which the program resides; and 3) the personnel and patients that provide and receive the program [13-15]. With ample description of the key elements that are appropriately analyzed and found effective, health care providers can then reproduce and modify elements within their own practice.

### Purpose

In anticipation of the changing Medicare payment structure and desire to improve outcomes within our medical system, we designed an intervention in late 2008 and implemented it in 2009 to reduce readmissions for HF patients that had identifiable and reproducible elements. We implemented and evaluated a disease management intervention that included Teaching and Education, prompt follow-up Appointments, Consultation for support services, and Home follow-up phone calls (TEACH-HF), to reduce all-cause rehospitalization rates in HF patients within 30 and 90 days of hospital discharge. Therefore, the objective of this study was to evaluate the effect of a disease management intervention on rehospitalization rates in hospitalized HF patients. More specifically, the aim was to analyze the readmission rates and cost of the TEACH-HF intervention implemented in 2010 - 2012 compared to 2007 - 2008 before TEACH-HF was implemented.

### Educational model

The educational model used in this study is called “teach-back”, although the term teach-back was applied to this model after it was first described [16]. The concept underpinning the method of teach-back education involves teaching patients key concepts and then asking patients to restate information that has been presented to them. Information is presented in plain language and is based on principles of health literacy. During teach-back, gaps in knowledge are clarified allowing the nurse to build on the patients’ baseline knowledge. Ensuring recall and comprehension is especially important for patients with varying levels of health literacy and chronic health conditions such as HF because of the complex treatment regimens, medication schedules, and need for self-monitoring for changes in health status [16].

## Methods

### Study design

This study was a prospective cohort design with a historical comparison group. This study was approved by the Institutional Review Board and the need for subject consent was waived (CHR #11-07783). Individual consent for participation was not required as all patients received this patient education as part of their usual care. The TEACH-HF intervention was implemented in January 2009. Participants admitted January 1, 2007 to January 1, 2008 (pre-teach-HF usual care cohort, n = 548) were compared to participants admitted from January 1, 2010 to January 1, 2012 (post-teach-HF intervention cohort, n = 485) when the intervention was fully in place.

### Setting and sample

The study took place at the University of California San Francisco Medical Center, a large urban academic medical center. Study participants included consecutive patients who were 65 years and older, admitted to the cardiology and medical services, and had a primary or secondary diagnosis of HF. Participants were excluded if they were admitted for less than 24 h or required advanced HF therapies (i.e. ventricular assist devices and heart transplantation).

The major outcome studied was rehospitalization. These data were obtained from the electronic medical record and from follow-up phone calls to patients and families at home after hospital discharge. Clinical and demographic data were retrieved from the electronic medical record and nurse coordinator database. Death data were retrieved using the social security death index (ancestry.com) through June 2012 or 6 months after hospital admission [17]. Death data were also verified by obituaries and family phone calls. Cost data were obtained from the Patient Safety and Quality Department at our hospital.

### Procedure

The two nurse coordinators identified appropriate patients from admission lists 7 days per week. When a patient was identified as eligible, the intervention began within 24 h of admission if the patient was stable. The intervention began with teaching and patient education and each intervention component is described below. The TEACH-HF intervention encompassed four key elements: 1) Teaching and patient Education; 2) prompt follow-up Appointments for within 7 days of hospital discharge; 3) Consultation for additional services including palliative care, case management, physical therapy, and home health, and 4) Home follow-up telephone calls within 7 days of discharge.

### TEACH-HF intervention components

#### Teaching and Education in the hospital

Two nurse coordinators provided all of the patient and family teaching. The education was focused on four focus areas: medications, self-monitoring skills, diet modification and warning signs for action. Specific teach-back questions were developed in relation to these four focus areas (Table 1). All patients received information on all four focus areas with the exception of patients who had end stage renal disease. We did not educate renal disease patients to weigh themselves daily. The nurse coordinators learned the teach-back method of education through the Institute for Healthcare Improvement (IHI) [18]. Family members and caregivers were included in the educational sessions when available and willing to participate. The educational sessions were provided daily from admission until discharge. Patient handouts corresponded to each teach-back focus area using materials from the American Heart Association and the IHI (available in English, Spanish, Russian, and Chinese). Medication reconciliation took place on admission and at phone follow-up.

**Table 1:** Teach-Back Questions

Teach-back question	Area of focus
What is the name of your water pill?	Medications
How much weight gain would you want to report to your MD?	Self-monitoring skills
What high-salt foods do you need to avoid/be aware of?	Diet modification
Please name 3 - 4 symptoms or warning signs of when you want to call the MD?	Warning signs for action

At the conclusion of the teaching each patient was asked four teach-back questions.

Emphasis was placed on development of self-management skills and patient empowerment to prompt action when necessary. The patient handouts were also provided to the home health nurses, nurse practitioners, clinic staff, and nursing home staff, to ensure that patients received the same education throughout the continuum of care.

#### Follow-up appointments

Clinic appointments within 7 days of hospital discharge for patients with a primary diagnosis of HF were provided as a way to improve the transition to home (patients with a secondary diagnosis of HF had clinic appointments requested within 7 - 14 days). If a patient was deemed “high-risk” for readmission, defined as two or more admissions within the past 12 months, had a complicated medical regimen, or was unsuccessful with teach-back, an appointment was made with either the cardiologist or nurse practitioner in the advanced heart failure clinic. A “hand-off” email was also sent to the referring provider regarding patient concerns. Additionally, a referral to the geriatrician for a home visit was made if patients met any of the following criteria: readmission in the past year; dementia, depression, anxiety or low health literacy; concerns for insufficient care support or caregiver burden; palliative care needs; history of poor medication compliance or missed appointments; and/or homebound, frail, and/or debilitated state. The geriatrician visited the patient within 48 h of discharge and ongoing as needed (Table 2).

**Table 2:** Sociodemographic and Clinical Variables (N = 1,033)

Variable	Usual care pre-TEACH (2007 - 2008) (n = 485)	Intervention post-TEACH (2010 - 2012) (n = 548)
Age (years), mean ± SD	80.1 ± 8.2	80.2 ± 8.3
Female gender, % (n)	53 (257)	54 (296)
Smoking in the past year, % (n)	5 (24)	4 (22)
Married/partnered, % (n)	41 (199)	40 (218)
Do not resuscitate orders, % (n)	13 (62)	17 (95)*
Length of hospital stay (index hospitalization) (days), mean ± SD	5.5 ± 7.0	5.6 ± 5.4

*P < 0.05.

#### Consultation

Inpatient consultations with the palliative care service, dietician, case manager or physical therapy were elicited to address unmet needs for symptom management, psychosocial and physical support. Palliative care consultations were obtained for all patients on their third HF admission within a 12-month period and when there was a clinical determination of need. The nurse coordinators completed End-of-Life Nursing Education Consortium training to initiate goals of care discussions [19]. Physical therapy and dietician consultations were routinely ordered by both physicians and the HF program coordinators if patient assessment revealed functional difficulties. Home health care nurse visits were recommended for all patients who qualified to provide on-going HF education, self-management skills, and medication reconciliation.

#### Home follow-up calls

Home follow-up phone calls took place within 5 - 7 days of hospital discharge to reinforce patient education and inquire about patient problems. The two nurse coordinators provided all home follow-up phone calls. Teach-back questions were again asked and patients were reeducated when necessary. Home services, medications, symptoms and any other concerns were discussed.

### Fidelity of the intervention

The TEACH-HF program was led by two expert HF nurse coordinators funded specifically for this program at 1.6 full time equivalents. The nurse coordinators, an advanced practice nurse and a certified HF registered nurse organized and provided the key parts of the intervention. The nurse coordinators learned the teach-back method of education through the IHI and with materials found on the IHI website [18]. This approach ensured the consistency of information presented to patients along with handouts. The time spent teaching, topics covered, and who was present during the teaching, were documented. This process made retrieval of data reliable and served as a prompt to cover all education topics with all patients.

They invited key staff and stakeholders to join the HF readmission reduction team including: the administrative director of the cardiac services, the medical director of the HF program, nursing faculty, a geriatrician, the medical director of the palliative care program, a dietician, a physical therapist, case managers, home health care nurses, chaplains, skilled nursing facility nurse administrators, case managers, a cardiology clinical nurse specialist, a pharmacist, and nurse practitioners from the outpatient HF clinic.

### Statistical analyses

All data were analyzed using SPSS statistical software version 19.0 (IBM Corp., Armonk, NY, USA). To assess the effectiveness of the intervention, we compared patients admitted during the 2010 - 2012 time period (post-TEACH-HF intervention) to all HF patients 65 years and older, admitted in 2007 - 2008 (pre-TEACH-HF usual care). A researcher not involved with the patient education or intervention process independently completed the data analysis. We used descriptive statistics for demographic characteristics of all patients. We compared characteristics of the intervention and usual care patient cohorts using Chi-square for categorical variables and Student’s *t*-test for continuous variables. An independent sample Mann-Whitney U test was utilized when comparing non-parametric data such as total number of hospital visits, as these data violated the assumptions of parametric tests. *Post hoc* contrasts with Bonferroni correction were performed using Chi-square tests in relation to discharge disposition. Survival analyses were completed using Kaplan-Meier probabilities with time to death within 30 and 90 days after the index hospitalization for patients discharged alive as the outcome variable. Rehospitalization was analyzed via the Cox proportional-hazards regression with time to rehospitalization event as the outcome variable. Alpha levels were pre-set at P < 0.05 and confidence intervals (CIs) were set at 95% with data presented as means ± standard deviations where appropriate.

We removed the patients who died during their first index admission prior to the data analysis to accurately determine the correct hospital readmission rate. Inpatient hospital costs were examined using the number of hospital bed days saved (or available beds for other patients), calculated as the number of admissions avoided within 30 days multiplied by the average length of stay (LOS). We also calculated the cost savings associated with reduced bed days by multiplying bed days saved by the average daily direct cost of one hospital day. For example, the average daily cost of one hospital day in 2012 was $1,876.00 (indirect and direct costs). Given that the hospital was not compensated for readmissions within 30 days, these cost savings all accrue to the medical center.

## Results

### Sample characteristics

Demographic and clinical characteristics (N = 1,033) of each group are listed in Table 2. The groups were similar with regard to characteristics such as age, gender, partnered/married, and length of hospital stay except that a higher percentage of patients in the post-TEACH intervention group had a do not resuscitate order (P = 0.04). The total number of hospital admissions per patient ranged from 1 to 11 and was not significantly different before or after the TEACH-HF intervention was started. In other words, hospital admission rates per patient did not decrease after the intervention.

### Clinical outcomes

**Figure 1:**
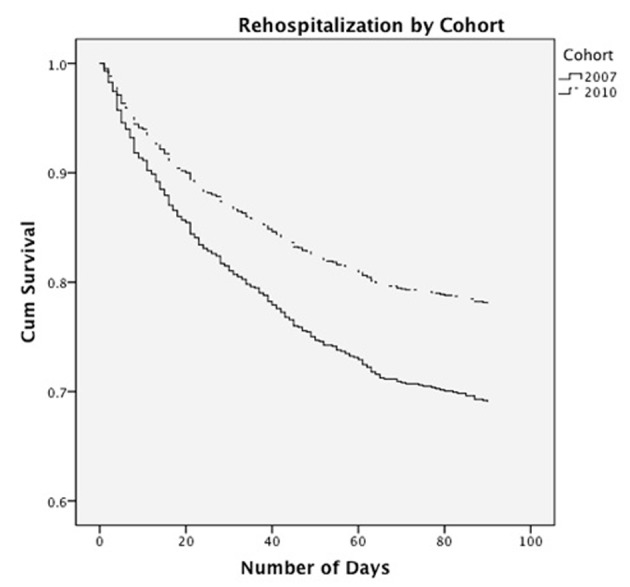
Rehospitalization curve by cohort.

**Figure 2:**
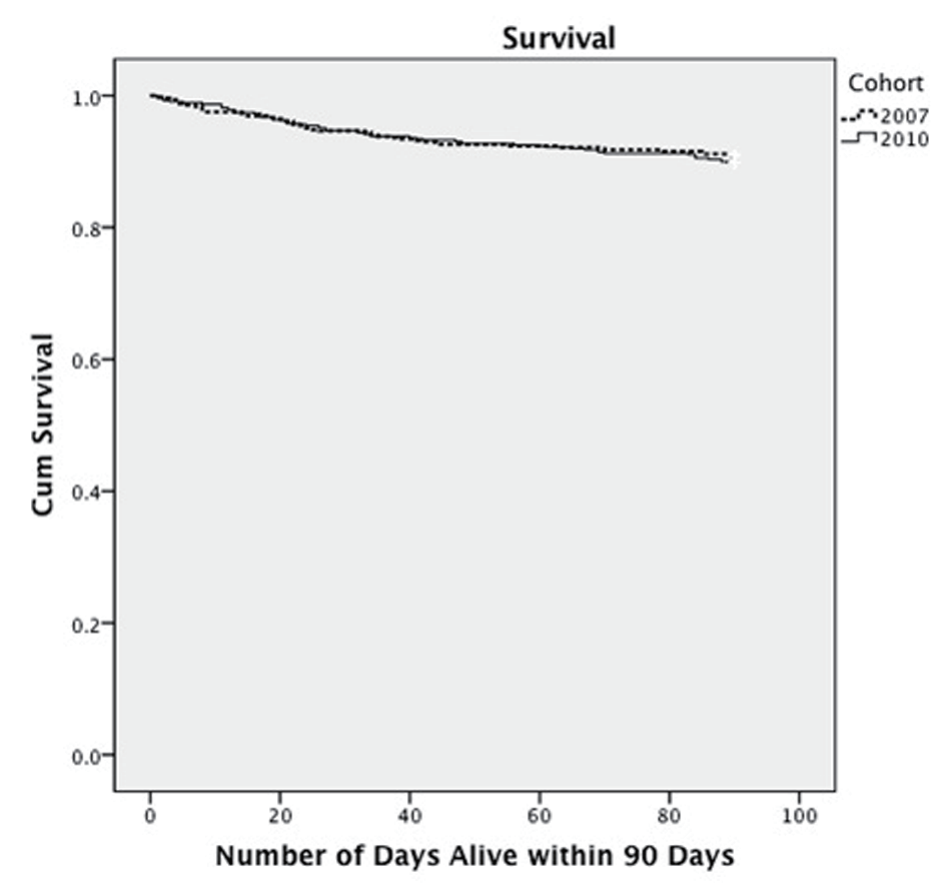
Survival curve by cohort.

Patients in the pre-TEACH usual care group had a 30-day readmission rate of 19% whereas in the post-TEACH intervention group it was 12% (P = 0.003) (Table 3). Similarly, patients in the pre-TEACH usual care group had a 90-day readmission rate of 30%, whereas in the post-TEACH intervention group, it was 19% (P = 0.001). When comparing data from 2010 - 2011 to 2011 - 2012, we found that the readmission rates in 2011 - 2012 were lower than in 2010 - 2011 (30-day readmission rate 8.7% vs. 15.5%; 90-day readmission rate 14.1% vs. 24.8% respectively, P < 0.03). Rehospitalization event risk was 1.5 times greater among patients in the pre-TEACH-HF usual care group (95% CI: 1.2 - 1.9; Fig. 1). Mortality rates at 30 and 90 days after discharge were similar in both the intervention and usual care groups reflecting a similar number of days alive after discharge (Fig. 2). The LOS did not change between the intervention and usual care groups. Finally, the average number of days to rehospitalization was 28 in the pre-TEACH usual care group and 29 in the post-TEACH intervention group revealing that although rehospitalization rates dropped, the amount of time to rehospitalization was the same, less than 30 days in each group.

**Table 3:** Rehospitalization and Disposition Outcomes (N = 1,033)

Variable	Usual care pre-TEACH (2007 - 2008) (n = 485)	Intervention post-TEACH (2010 - 2012) (n = 548)
Rehospitalized within 30 days of discharge, % (n) after removing patients who had died (n = 29 died; n = 519)	19 (93)	12 (68)**
		2010 - 2011: 15.5 (52) (n = 335) 2011 - 2012: 8.7 (16)* (n = 184)
Rehospitalized within 90 days of discharge, % (n) after removing patients who had died (n = 56 died; n = 492)	30 (147)	19 (102)**
		2010 - 2011: 24.8 (78) (n = 315) 2011 - 2012: 14.1 (25) (n = 177)
Died within 30 days of discharge, % (n)	5.3 (26)	5.3 (29)
		2010 - 2011: 5.4 (20) 2011 - 2012: 4.6 (9)
Died within 90 days of discharge, % (n)	9.1 (44)	10.2 (56)
		2010 - 2011: 10.9 (40) 2011 - 2012: 8.1 (16)
Discharged home with no services, % (n)	61 (296)	31 (161)***
Discharged with home health services, % (n)	16 (79)	49 (257)***
Discharged to skilled nursing facility, % (n)	18 (88)	15 (79)
Discharged to acute hospital, % (n)	0.8 (4)	2 (12)
Discharged to hospice, % (n)	3.7 (18)	3 (18) (n = 527)^§^

*P < 0.05, **P < 0.01, ***P < 0.001. ^§^Twenty patients with unknown disposition data.

### Components of the TEACH-HF intervention

During the TEACH-HF intervention, 492 patients received daily education during their hospital stay. Reasons patients did not receive teaching include: dementia/no caregiver (n = 18), patient readmission with education recently provided (n = 4), hospice care (n = 6), patient unavailable/undergoing diagnostic testing (n = 3), patient refused (n = 3), unknown reasons (n = 12), and skilled nursing facility residence (n = 13) where self-management skills are not possible. The daily mean time spent teaching was 38 ± 15 min. We found no difference in the amount of time spent teaching patients who were rehospitalized versus not rehospitalized at 30 or 90 days after discharge.

Overall, 91% of patients had an appointment arranged after their hospital discharge. The geriatrician visited 10% (n = 55) of patients at home after discharge. Inpatient consultations or referrals for palliative care, case management, physical therapy and dietary services were 5%, 54%, 82, and 24% respectively in the post-TEACH group (consultation and appointment data before the TEACH-HF intervention were not collected). Referrals to home health services after hospital discharge significantly increased from 16% to 49% (P < 0.0001) when comparing the pre- to post-TEACH groups (Table 3). Fewer patients were discharged home alone or with a family member without home health services in the post-TEACH group (P < 0.0001), while discharges to skilled nursing facilities remained unchanged (P < 0.11). Home follow-up phone calls within 1 week of discharge took place with 100% of patients in the post-TEACH group (after excluding patients who were not eligible for a phone call). Reasons that patients (n = 171) did not receive a phone call include: readmitted (n = 13), transferred (n = 2), discharged to skilled nursing facility (n = 82), no answer/disconnected (n = 12), died/hospice program (n = 14), or other (n = 23).

### Inpatient costs

Inpatient hospital costs were examined using the number of bed days saved (or available beds for other patients) in relation to our readmission rates. In the pre-TEACH usual care cohort 93 patients were readmitted within 30 days (2007 - 2008) with an average LOS of 5.5 days (Table 2). During the first year of the TEACH-HF intervention (2010), 52 patients were readmitted within 30 days with an LOS of 5.6 days. The difference in bed days between 2007 and 2010 was 218 bed days (93 - 52 = 39 admissions; 39 admissions × 5.6 days = 218 bed days). During the second year of the intervention (2011 - 2012), 16 patients were readmitted within 30 days. The difference between 2007 - 2008 and 2011 - 2012 was 431 bed days (93 - 16 = 77; 77 admissions × 5.6 days = 431 bed days) available for other patient admissions. A total of 649 bed days were saved in 2 years, an average of 324 bed days per year. A reduction of 324 unreimbursed bed days per year would either save nearly $640,000 per year in operating costs and provide beds with billable codes (i.e. permit operational charges to be submitted).

Costs incurred by the TEACH intervention were almost entirely personnel related with the bulk of the cost from the nurse coordinators. These personnel costs included salary and fringe benefits for one full-time and one 60% registered nurse position. These salary costs are fixed by the union that represents the nurses at our facility. Costs of patient materials were minimal. Costs were also associated with each member of the HF team as their time was donated. Additional outpatient costs (but not calculated) include outpatient visits with the primary care physician, nurse practitioner, cardiologist, geriatrician and home health services when utilized.

## Discussion

The TEACH-HF intervention was associated with a significant reduction in all-cause hospital readmissions within 30 and 90 days without changes in mortality rates (or days alive) when compared to the pre-TEACH HF cohort. The reduction in hospital readmission rate improved over time and made additional patient beds available for revenue. This study is unique in three ways: 1) a health literacy-appropriate method of teaching was used, 2) a comprehensive support system was provided, and 3) services already in place were utilized rather than creating a new resource intensive structure for HF patients at high risk for hospital readmission. Notably, despite the reduction in hospital readmission rates, the average readmission time was less than 30 days in both groups.

Although nearly 90% of hospitals have a written objective of reducing readmissions for HF patients, the implementation of recommended methods vary widely [20]. We developed a multidisciplinary approach that spanned across the continuum of care. We integrated several proven methods for improving care provided by trained nurses whose sole job was to implement this intervention. We provided patient education daily and again 5 - 7 days after hospital discharge. Data in relation to patient education alone and reduction in hospital readmission are mixed [21, 22], although guidelines from the Heart Failure Society of America recommend patient education for all HF patients [23]. Investigators found significant reductions in HF readmissions after a 1-h personal education session, but assessment of learning was not reported [24]. While teach-back has not been tested against other patient education methods, investigators have demonstrated that teach-back is associated with knowledge retention in HF patients [25]. However, teach-back by itself was not associated with a reduction in all-cause rehospitalization rates [25]. Kripilani et al demonstrated that teach-back was an effective method to assess knowledge retention for research participation in a study of low-literacy adults [26]. While knowledge does not insure adherence to prescribed therapies, knowledge is an essential first step for patients to engage in self-management.

Early follow-up appointments after hospital discharge were demonstrated to reduce the risk of 30-day hospital readmissions in a large study of HF patients [27]. Although we did not provide patient follow-up appointments for 100% of the patients, we did substantially increase the appointment rates. A concerted effort was made to provide a 7-day follow-up appointment for all high-risk patients and this may have contributed to our readmission reduction. We had a higher rate of referral for home health services and this may have influenced our readmission rates and increased overall outpatient costs. However, these costs are much lower than inpatient hospital days. In addition, some older HF patients received a visit at home by our geriatrician and may also provide a safety net that is needed to ensure a smooth transition to home. Most importantly, medication reconciliation took place during the hospital follow-up visit either with the home care nurse or the health care provider.

Consultation for physical therapy, palliative care, case management, dietician and home care services were provided to support additional needs. We found a significant increase in home health care referrals that identified problems at home. Home follow-up phone calls by the HF nurse coordinators also served as early alerts to problems. When problems were identified, communication with either the patients’ primary care provider or cardiologist took place.

While each patient did not receive all components of the TEACH-HF intervention, every patient did receive at least several components. All patients received teaching and education, more than 90% had early appointments arranged, and almost half had home health referrals. Consultations varied based on patient needs. A meta-analysis that examined interventions aimed to reduce 30-day readmission rates determined that most studies tested a single component intervention and that no single intervention alone was associated with a reduced risk for HF readmissions [10]. Twelve studies tested three or more interventions, similar to this study. However, a limited description for these bundled interventions was provided making it impossible to determine the effect of an individual intervention. Our intervention was also bundled, but each component was discrete and described. Since HF is a complex problem, it seems logical that a multi-focal intervention that addresses social issues would be needed to change patient outcomes [28].

Reducing hospital readmissions has enormous financial and patient care implications. Recognizing and demonstrating the financial consequences of a disease management program are an essential step if HF programs are to remain sustainable. This change could result in significant cost savings and revenue loss (if the beds are left empty) or potential revenue engines if these beds are filled with patients bearing reimbursable medical conditions. The potential cost savings of $640,000 per year is a conservative estimate as the cost of intensive care unit days was not included in the bed day cost estimate. Other investigators have had equivocal results when examining costs in relation to patient outcomes [11, 29, 30].

Several limitations of our study must be acknowledged. First, the lack of a concurrent control group prevented a direct comparison to patients who did not participate in the TEACH-HF intervention in the same time period. However, the Medicare changes did not come into effect until October 2012 and there were no significant changes in medical practice for HF that could explain the results, such as new drugs. Second, our findings can only be applied within the context of our setting and application of the key components of our intervention may not provide the same outcomes elsewhere. However, our patients with HF averaged 80 years of age, typical of patients with HF and we excluded patients that required advanced interventions and thus it is likely that our patients are similar to HF patients at other hospitals. Third, we did not account for possible admissions to other hospitals during the study observation period. Imbalances in hospitalizations elsewhere in 2007 - 2008 compared to 2010 - 2012 might possibly affect our data and conclusions significantly. Finally, we were unable to conduct a complete cost analysis that included the outpatient setting as these data were not available to us. However, all other services provided were standard interventions that could be provided to all patients and covered by insurance including Medicare. Despite these limitations, these data reflect a “real world” practical view of an intervention to reduce HF readmissions.

### Conclusion

The TEACH-HF intervention was associated with fewer all-cause hospital readmissions within 30 and 90 days without a change in mortality rates. This reduction was in comparison to the HF patients admitted prior to the intervention who were similar in age, gender and length of index hospital stay. We utilized a health literacy-appropriate multidisciplinary method of patient teaching and enhanced services that utilized hospital resources already in place, rather than creating a new service intensive infrastructure. Reduction in hospital readmissions improved over time and made additional patient beds available for revenue and other patients. The four key components within this intervention can provide a framework for investigators to apply to future HF interventions. An HF disease management intervention that includes teaching and education, prompt follow-up appointments, consultation for support services, and home follow-up phone calls and is health literacy-appropriate, may be an effective way to reduce all-cause rehospitalization rates. This study may help launch further research to provide a potential solution to the challenge in hospital readmissions.
